# Mutational analysis of *BRCA1*/2 in a group of 134 consecutive ovarian cancer patients. Novel and recurrent *BRCA1*/2 alterations detected by next generation sequencing

**DOI:** 10.1007/s13353-014-0254-5

**Published:** 2014-11-01

**Authors:** Magdalena Ratajska, Magdalena Krygier, Maciej Stukan, Alina Kuźniacka, Magdalena Koczkowska, Mirosław Dudziak, Marcin Śniadecki, Jarosław Dębniak, Dariusz Wydra, Izabela Brozek, Wojciech Biernat, Ake Borg, Janusz Limon, Bartosz Wasąg

**Affiliations:** 1 Department of Biology and Genetics, Medical University of Gdansk, Debinki 1, 80-210 Gdansk, Poland; 2 Department of Gynecologic Oncology, Gdynia Oncology Center, Gdynia, Poland; 3 Department of Gynaecology, Gynaecological Oncology and Gynaecological Endocrinology, Medical University of Gdansk, Gdansk, Poland; 4 Department of Pathomorphology, Medical University of Gdansk, Gdansk, Poland; 5 Department of Oncology, CREATE Health Strategic Center for Translational Cancer Research, Skane Department of Oncology, Lund University, Skane University Hospital, Lund, Sweden

**Keywords:** *BRCA1*, *BRCA2*, Mutations, Next generation sequencing, Ovarian cancer, PARP inhibitors

## Abstract

The importance of proper mutational analysis of *BRCA1/2* in individuals at risk for hereditary breast and ovarian cancer syndrome is widely accepted. Standard genetic screening includes targeted analysis of recurrent, population-specific mutations. The purpose of the study was to establish the frequency of germline *BRCA1/2* mutations in a group of 134 unrelated patients with primary ovarian cancer. Next generation sequencing analysis revealed a presence of 20 (14.9 %) mutations, where 65 % (*n* = 13) were recurrent *BRCA1* alterations included in the standard diagnostic panel in northern Poland. However, the remaining seven *BRCA1/2* mutations (35 %) would be missed by the standard approach and were detected in unique patients. A substantial proportion (*n* = 5/12; 41 %) of mutation-positive individuals with complete family history reported no incidence of breast or ovarian cancer in their relatives. This observation, together with the raising perspectives for personalized therapy targeting *BRCA1/2* signaling pathways indicates the necessity of comprehensive genetic screening in all ovarian cancer patients. However, due to the limited sensitivity of the standard genetic screening presented in this study (65 %) an application of next generation sequencing in molecular diagnostics of *BRCA1/2* genes should be considered.

## Introduction

*BRCA1* and *BRCA2* germline mutations are associated with high penetrance for both breast and ovarian cancer (Miki et al. 1994; Wooster et al. 1995). The overall prevalence of *BRCA1/2* alterations in general population varies considerably among different ethnic groups, with respect to specific founder mutations. In 2000, three founder alleles of *BRCA1* (c.5266dup, c.181T>G, c.4034delA) were reported in Polish families with a strong aggregation of breast or ovarian cancers and subsequently incorporated into the standard genetic screening panel (Gorski et al. 2000). Further investigations were expanded in a large population of unselected female breast cancer patients (Brozek et al. 2011; Gaj et al. 2012; Lubinski et al. 2006; Szwiec et al. 2014) as well as in consecutive ovarian cancer series (Brozek et al. 2008; Majdak et al. 2005; Menkiszak et al. 2003). A relatively high germline mutation frequency at the level of 13.5 % for *BRCA1* and 13.9 % for *BRCA1/2* among cases with unselective primary ovarian carcinoma has been reported in studies by Menkiszak et al. (2003) and Brozek et al. (2008), respectively. Albeit both studies confirmed the strong founder effect for *BRCA1* 5266dup and c.181T>G alterations, the latter reported other recurrent *BRCA1/2* mutations in the group of Polish patients with ovarian cancer.

In line with the recommendations of the American Society of Clinical Oncology testing for recurrent *BRCA1* mutations is required in each case of ovarian cancer in the Polish population (1996). According to the reports of the latest clinical trials patients with *BRCA* mutations and recurrent serous ovarian carcinoma may benefit from specific therapies targeting *BRCA* signaling pathways (Audeh et al. 2010; Fong et al. 2009, 2010; Ledermann et al. 2012, 2014). For the purpose of the most effective treatment as well as genetic counseling and prophylactic strategies for patients and their families, mutational analysis of *BRCA1* and *BRCA2* needs to be highly sensitive and cost-efficient.

The aim of this study was to establish the frequency of germline *BRCA1/2* mutations in consecutive ovarian cancer series from northern Poland. Additionally, it was of our interest to investigate whether an application of next generation sequencing can significantly improve *BRCA1/2* mutation detection rate and subsequently effectiveness of further prophylactic and treatment strategies.

## Materials and methods

### Study material

The study comprises 134 unselected ovarian cancer patients who were referred to the University Hospital in Gdansk and the Red Cross Hospital in Gdynia between 2012 and 2013. Within the studied group 77.6 % (*n* = 104/134) of patients were diagnosed with serous ovarian cancer, average age at diagnosis was 60.8 (24–87) years. Informed consent was obtained from all of the patients and the study was approved by the medical review board of Medical University of Gdansk.

### DNA extraction

Genomic DNA was extracted from the whole blood using red-blood-cells lysis buffer followed by the standard phenol-chloroform procedure as described elsewhere.

### Mutational analysis

*BRCA1* and *BRCA2* mutation screening was performed using the *BRCA* MASTR assay v1.2 (Multiplicom, Niel, Belgium) followed by MiSeq targeted re-sequencing at minimum of 99x coverage (Illumina Inc.). The cut-off of 20 % was applied. The analysis was performed with Illumina Variant Studio Software (Illumina Inc.) and Geneious Software (Biomatters Ltd). Presence of the *BRCA1/2* mutations detected by NGS analysis was confirmed by PCR followed by bi-directional Sanger sequencing (ABI PRISM 3130, Life Technologies, Inc.).

## Results

In the group of 134 patients with unselected primary ovarian cancer, pathogenic *BRCA1* or *BRCA2* mutations were found in 20 individuals (14.9 %). In addition, two variants of unknown significance were detected (*BRCA1*: c.301+7G>A; *BRCA2*: c.9486_9488del). Among the *BRCA1/2* positive cases, 16 carried *BRCA1* and four *BRCA2* mutation, which accounts for 80 and 20 %, respectively. Thirteen alterations (*n* = 13/20; 65 %) were recurrent *BRCA1* mutations included in the standard genetic screening panel used in northern Poland, as previously reported (Ratajska et al. 2008). The remaining seven mutations (*n* = 7/20; 35 %), three located in *BRCA1* and four in *BRCA2* are not included in the standard targeted mutation analysis, which overall gives seven out of the 20 mutation positive cases (35 %). All these mutations were detected in unique patients. Detailed clinical, histopathological and molecular data of *BRCA1/2* positive patients are presented in Table 1.

**Table 1:** Clinical, histopathological, and molecular data of the ovarian cancer patients with *BRCA1/2* mutation

No.	Case no	Exon/intron	Mutation in corresponding cDNA^a^	Predicted amino acid sequence	Mutation type^b^	RS number^c^	Age (years)	FIGO stage	Histology	Family history^d^
BRCA1 gene										
1	115	5	c.181T>G (300T>G)	p.Cys61Gly	M	28897672	36	IIIC	serous	negative
2	296						54	IIIC	serous	positive
3	22	11	c.3700_3704del (3819del5)	p.Val1234Glnfs*8	F	80357609	61	IIIB	serous	unknown
4	38						43	IIIC	serous	negative
5	66						52	unknown	serous	unknown
6	50	20	c.5266dup (5382insC)	p.Gln1756Profs*74	F	397507246	52	IIIC	serous	positive
7	108						63	unknown	serous	unknown
8	138						65	unknown	serous	positive
9	314						46	IIIC	endometrioid	positive
10	323						37	IIIC	serous	positive
11	368						60	unknown	serous	unknown
12	374						66	unknown	serous	unknown
13	378						36	unknown	serous	unknown
14	78	9	c.594-2A>C	r.[=;594_670del] p.Val179Cysfs*3	F	80358033	24	unknown	serous	unknown
15	85	10	c.1793T>A	p.Leu598*	N	80357118	43	unknown	endometrioid	negative
16	95	13	c.4357+2T>G;	r.[=;4186_4357del] p.Arg1377Tyrfs*2	F	80358152	45	IIIC	serous	negative
BRCA2 gene										
17	3	11	c.3975_3978dup	p.Ala1327Cysfs*4	F	397515636	45	IV	serous	positive
18	93		c.2808_2811del	p.Ala938Profs*21	F	80359351	82	unknown	serous	positive
19	103		c.5042_5043del	p.Val168Glufs*7	F	80359478	62	IIIC	serous	negative
20	87	14	c.7180A>T	p.Arg2394*	N	80358946	67	IIIC	endometrioid	unknown

^a^Mutation type according to the HGVS nomenclature; HGVS, Human Genome Variation Society. Nomenclature commonly used is shown in brackets

^b^F, frameshift; M, missense; N, nonsense

^c^A reference SNP number

^d^a positive family history was determined when at least one family member with breast/ovarian cancer was present besides the patient

Out of the 20 *BRCA1/2* positive patients, 17 were diagnosed with ovarian serous adenocarcinoma (85 %), whereas the other three with adenocarcinoma endometroides (15 %). Overall, the frequency of *BRCA1/2* mutations was 40 % (*n* = 9/22) in the group diagnosed before or at age of 50 and approximately 10 % among patients older than 50 years (*n* = 11/112). The mean age of cancer diagnosis was 52 (24–82) years in the group of mutation carriers and 62.3 (27–87) years in patients with *BRCA1/2* wild-type. Finally, detailed family history was available only in 60 % (*n* = 12/20) of *BRCA1/2* positive patients, of whom in five individuals it was negative for breast/ovarian cancer in first and second degree relatives (41 %).

## Discussion

The percentage of *BRCA1/2* mutations in unselected ovarian cancer patients identified in the current study is comparable to that previously reported (14.9 vs 13.5 and 13.9 %) (Brozek et al. 2008; Menkiszak et al. 2003). The worldwide prevalence of *BRCA1/2* mutations in consecutive ovarian cancer series is estimated at 5 to 15 % and varies markedly depending on the population’s ethnic background (Berchuck et al. 1998; Malander et al. 2004; Risch et al. 2001). Effective and cost-efficient targeted mutation analysis is widely performed in patients with familial history of breast and ovarian cancer, including population-specific founder mutations. Since the implementation of genetic screening panel covering the three founder mutations in 2000, there have been subsequent reports on the prevalence of other recurrent *BRCA1/2* mutations in Poland (Brozek et al. 2008; Gaj et al. 2012; Gorski et al. 2004; Perkowska et al. 2003; Ratajska et al. 2008; Szwiec et al. 2014). Based on these reports, the standard testing panel has been expanded to five *BRCA1* alterations in northern Poland (Ratajska et al. 2008). Furthermore, more recently, Szwiec et al. (2014) proposed to test six *BRCA1* mutations in a group of women diagnosed with breast cancer at the age of 50 or below.

It is widely accepted that the differences in frequency and spectrum of *BRCA1/2* mutations may result from an application of different molecular techniques or ethnic diversity even within the same country (Brozek et al. 2011; van Der Looij et al. 2000).

To validate usefulness of targeted assays in screening for *BRCA1/2* mutations, we performed a next generation sequencing in the group of 134 patients with unselected ovarian cancer. Based on the results we conclude that the sensitivity of genetic screening covering the three founder *BRCA1* mutations (c.5266dup, c.181T>G, c.4034delA) is established at 50 % (only ten out of 20 mutations). Expanded panel, comprising two additional recurrent mutations in *BRCA1* (c.3700_3704del and c.68_69delAG) increases detection rate to 65 %.

In the present study fairly high frequency of previously selected mutations was confirmed. c.5266dup was the most common *BRCA1* alteration detected in the studied group (*n* = 8/13; ∼62 %). The second most common *BRCA1* mutation was a frame shift deletion c.3700_3704del (*n* = 3/13; 23 %). The c.3700_3704del mutation is frequently detected in the Caucasian populations and has been reported as one of the three most prevalent alterations in north-eastern and south-central Poland, along with the c.5266dup and c.181T>G (Brozek et al. 2008, 2011; Gaj et al. 2012; Ratajska et al. 2008; Szwiec et al. 2014). Furthermore, a relatively low frequency of the other two founder mutations, c.4034delA and c.68_69delAG in our region denotes heterogenity of *BRCA1* alterations in Polish patients with ovarian cancer.

The remaining seven mutations detected in *BRCA1* and *BRCA2* genes were identified only in unique patients. However, all detected genetic variants have been previously reported in the Breast Cancer Information Core (BIC) database (Szabo et al. 2000).

Interestingly, within this study a relatively high proportion of deleterious *BRCA2* mutations (20 %) was observed. For instance, a c.2808_2811del deletion identified in an 82-year old patient is a recurrent mutation, frequently detected in non-Ashkenazi breast/ovarian cancer patients and described to have multiple origins (Infante et al. 2013). The other *BRCA2* genetic variants detected in the current study, although less frequent, were also previously reported (Szabo et al. 2000).

A substantial percentage of *BRCA1/2* positive patients diagnosed after the age of 60 (*n* = 8/20) is consistent with the previous reports, indicating that the incidence of ovarian cancer in older age does not exclude *BRCA* germline mutation, especially as far as the *BRCA2* gene is concerned (Brozek et al. 2012; Risch et al. 2001). Limited significance of age at diagnosis as well as patient’s family history justifies genetic screening for the founder *BRCA1/2* mutations in all ovarian cancer patients. However, according to the current study, as many as 35 % of mutation positive individuals can be missed in the routine screening. This proportion can be even higher after taking into account possible large rearrangements in *BRCA* genes that were not examined in the present study. Though, as previously reported (Ratajska et al. 2008; Rudnicka et al. 2013) these *BRCA1/2* mutations are relatively rare (3.7 and 4.8 % of all *BRCA1* alterations, respectively) in the Polish population, we cannot entirely exclude their occurrence. Moreover, a deletion comprising exon 17 has been detected in two unrelated Polish high-risk breast and ovarian cancer families (Rudnicka et al. 2013).

The importance of mutation detection, apart from genetic counselling and prophylactic management arises in the considerance of recent clinical trials, with the implementation of poly (ADP-ribose) polymerase 1 (PARP1) inhibitors. PARP1 is a member of chromatin-associated polymerases involved in posttranslational ADP-ribosylation and DNA strand breaks repair (El-Khamisy et al. 2003; Lindahl et al. 1995). It has been reported that tumor cells with *BRCA1/2* mutation, since their deficiency in homologous recombination, are markedly sensitized to the PARP inhibition, resulting in chromosomal instability and consequent apoptosis (Bryant et al. 2005; Farmer et al. 2005). According to the results of phases 1 and 2 trials with PARP1 inhibitor Olaparib, *BRCA* mutation positive patients with platinum-sensitive relapsed serous ovarian cancer respond preferentially to PARP inhibition, with a significant reduction in risk of disease progression in comparison to patients with wild-type *BRCA* (Audeh et al. 2010; Fong et al. 2009, 2010; Ledermann et al. 2012, 2014). These observations raise promising perspectives for personalized therapy in *BRCA* carriers and further emphasizes the necessity for proper mutational analysis of *BRCA1/2* in a group of ovarian cancer patients.

## Conclusions

In conclusion, we propose routine genetic screening for at least five recurrent *BRCA1* mutations (c.5266dup, c.181T>G, c.3700_3704del, c.68_69delAG, c.4034delA) in all ovarian cancer patients in the Polish population. In addition, in the negative cases for above-mentioned alterations analysis of the entire *BRCA1/2* genes with an application of new generation sequencing should be performed. Unfortunately, given the potential costs of such expanded diagnostics it will probably need to be limited to the selected group of patients with specific clinical and histopathological characterization. To establish recommendations for the entire *BRCA1/2* genes mutational analysis further studies in larger ovarian cancer series with detailed family history are required. Moreover, based on the results we confirm that next generation sequencing is a sensitive, reliable tool which can be applied in molecular diagnostics of *BRCA1/2* genes in a group of selected patients.
